# Infectious Diseases in Dublin

**Published:** 1896-10-17

**Authors:** 


### INFECTIOUS DISEASES IN DUBLIN.

In his introductory address delivered at St. Vincent's Hospital, Dublin, on October 6th, Dr. M'Hugh described the existing accommodation for infectious diseases in that city as "in need of radical reform," and, "as a whole, utterly inadequate to meet the requirements of the case." In addition to the Cork Street and the Hardwicke Hospitals, which take rank as special fever hospitals, there seem to be fever departments in no less than six of the general hospitals and in one private hospital, so that in a city of no very considerable dimensions the treatment of fever is carried on at eight or nine different centres, many of which are situated in the midst of thickly populated districts. It would seem impossible for the various kinds of fever to be separated from one another, and from other non-infectious cases, where the fever department consists merely of the wing of a building which admits all classes of medical and surgical diseases, or at most of a small detached block in close connection with it. In these institutions it appears that the visiting medical staff is the same both for the general medical and the fever wards; the resident medical officers and pupils, and the students attending, pass freely from one section to another; the nursing administration is common to both, and it is not too much to say that the principle of isolation is practically ignored. It certainly is a striking thing that the people of Dublin should not only put up with, but actually defend, a state of affairs so different to what we aim at on this side of the channel, and in view of the growing demand for hospital treatment for all kinds of infectious diseases, which we see here, it is not without interest to observe that in the various discussions which have taken place in Dublin in regard to the erection of special hospitals for such cases, medical and sanitary opinion has by no means unanimously supported the proposal, and that many leading men have expressed themselves strongly in favour of continuing the present system. The influence of isolation, as we understand the term, is clearly not accepted in Dublin as the indisputable axiom which it is held to be by many English sanitarians. Yet they claim there, as we do here, that zymotic disease is on the wane!

